# Doing more with less: Genomic quasi-G-primes differentiate septic from healthy patients

**DOI:** 10.1371/journal.pone.0341828

**Published:** 2026-02-06

**Authors:** Congzhou M. Sha, Michail Patsakis, Ioannis Mouratidis, Xiaoyuan Wei, Taejung Chung, Jasna Kovac, Ilias Georgakopoulos-Soares

**Affiliations:** 1 Department of Molecular and Precision Medicine, Institute for Personalized Medicine, The Pennsylvania State University College of Medicine, Hershey, Pennsylvania, United States of America; 2 National Technical University of Athens, School of Electrical and Computer Engineering, Athens, Greece; 3 Department of Pharmacology and Toxicology, The University of Texas at Austin College of Pharmacy, Austin, Texas, United States of America; 4 Huck Institutes of the Life Sciences, The Pennsylvania State University, University Park, Pennsylvania, United States of America; 5 Department of Food Science, The Pennsylvania State University College of Agricultural Sciences, University Park, Pennsylvania, United States of America; 6 Department of Internal Medicine, Morsani College of Medicine, University of South Florida, Tampa, Florida, United States of America; Rutgers Biomedical and Health Sciences, UNITED STATES OF AMERICA

## Abstract

Sepsis is a life-threatening state of disseminated infection, and treatment requires knowledge of the organism responsible. The gold standard for sepsis diagnosis is blood culture, which requires days of growth. Next-generation sequencing has been proposed as an alternative; however, existing methods may lack sensitivity. In this work, we explore the idea of genomic quasi-G-primes, which are short DNA sequences specific to a single species within a group of relevant species. We first validated the genomic quasi-G-prime classification in controlled *Staphylococcus aureus* sequencing experiments, and then applied the same approach to blood-derived sequencing data from septic and healthy patients, where genomic quasi-G-prime profiles distinguished disease states. Our method is highly space-efficient, permitting fast classification on modest hardware and enabling it to outperform existing taxonomic classification approaches in this task.

## Introduction

Next-generation sequencing (NGS) is increasingly used in clinical practice, such as in tumor clinical oncology to guide cancer therapy [1], and to diagnose rare genetic diseases [2]. Multiple experimental methods are encompassed by NGS, and the term refers to the ability to sequence many short nucleotide sequences (RNA or DNA) in parallel, allowing for an unbiased view of all nucleotides present in a sample. One of the fundamental barriers to interpreting NGS data is assignment of nucleotide reads to specific species, posing a significant challenge in microbiomics and complicating the application of NGS to infectious disease.

**Fig 1:**
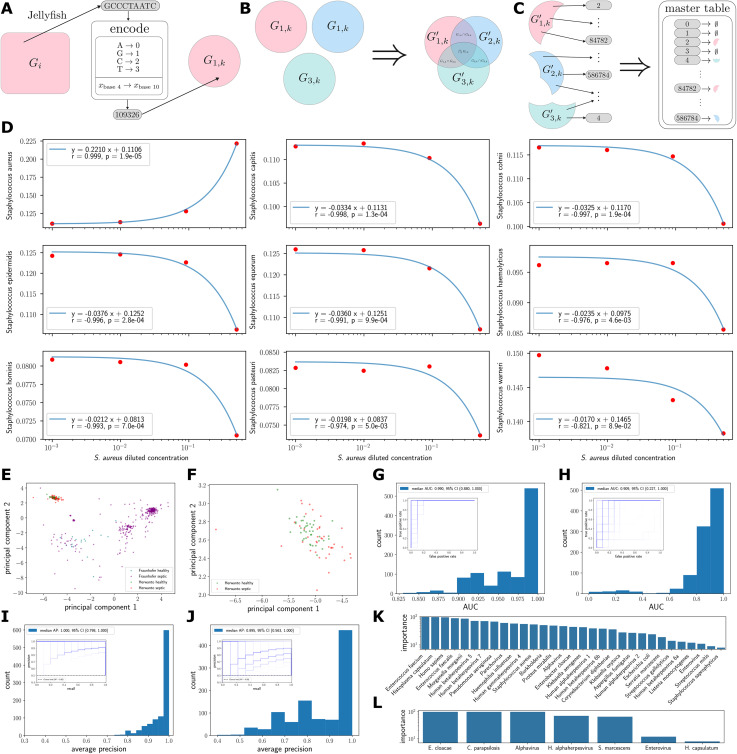
Graphical illustration of quasi-prime pipeline, with *in vitro* and clinical proof-of-concept. **A**. All kmers present in a genome ${𝐆}_{\text{1}}$ are encoded as integers and placed in a set ${𝐆}_{\text{1},k}$. **B**. Once kmer sets have been constructed for all genomes of interest, the quasi-prime sets ${𝐆}_{i,k}^{\prime }$ are determined through set operations. **C**. Now that the ${𝐆}_{i,k}^{\prime }$ are disjoint, we construct the mapping from quasi-primes to species. **D**. Proportion of reads assigned to each species as a function of the diluted DNA concentrations of *S. aureus*, along with linear regressions and 𝐫 and 𝐩 values. **E and F**. Principal component analysis of normalized read counts. The Herwanto data points occupy a distinct corner (**F**) of the space. **G and H**. AUC distributions for logistic regression of Herwanto and Fraunhofer, respectively. Underlying ROC curves are inset at low opacity to visualize the curve density. **I and J.** Average precision distributions for Herwanto and Fraunhofer, respectively. Underlying precision‒recall curves are inset at low opacity to visualize the curve density. **K and L**. are the most important species in classifying septic vs healthy patients for Herwanto and Fraunhofer, respectively.

Recently, the concept of genomic quasi-primes has been proposed to aid in organism identification and classification (Figs 1A-C) [3–5]. The essential insight is that not all sequences detected in a sample are equally informative, and one should focus on the subset of short sequences which are most likely to be unique and differentiate between species. In the absence of noise in the data, one would consider only those short sequences which occur in the genome of one species and not in any other, so that detection of such a sequence in a sample suggests that the species it belongs to is also present in the sample. In other words, such a sequence is highly specific, allowing the researcher to rule in the species. These sequences are not shared between any two or more genomes, resulting in the disjoint regions shown in Fig 1C. Note that in this work, we restricted our attention to quasi-primes for a specific set of bacterial, viral, and fungal species known to cause sepsis in humans.

Because NGS is also highly sensitive [6,7], if species-specific sequences are not detected, it is unlikely that the associated species is present at clinically relevant levels, allowing the researcher to rule out the species as the major infectious cause. The combination of high specificity and high sensitivity of NGS lends itself to identifying the causative organism in sepsis. In this work, we provide a proof of concept that genomic quasi-primes provide sufficient signal-to-noise ratio to identify *Staphylococcus aureus* in sequencing experiments and to classify septic versus healthy patients in clinical settings.

Unlike polymerase chain reaction (PCR), NGS is a hypothesis-free method of capturing the genetics of a sample. PCR relies upon curation of primers, which biases the sequences which are detected. In principle, NGS detects all nucleotide sequences present in the sample without preference. Although we use NGS data in this study, we note that identifying quasi-primes may also be useful in designing PCR primers to improve the sensitivity of PCR in detecting foreign genomes.

We will review the mathematical description of quasi-primes. We refer to a sequence of length k as a kmer. For a set of genomes $G=\{G_{\text{1}\;},G_{\text{2}},\cdots ,G_{n}\}$, let $G_{i,k}$ denote the set of all kmers in $G_{i}$. A **kmer quasi-G-prime of** $G_{i,k}$ is a sequence of length k that occurs only in $G_{i}$ and not in any other $G_{j}$. The addition of “G” in quasi-G-prime indicates that the quasi-primes were restricted to a small set of clinically relevant genomes, rather than in typical analyses of universal taxonomies.

Existing approaches to organism identification in sepsis rely on universal taxonomy classification (e.g., Kraken [8,9], Kaiju [10]) to classify reads, whereas clinically relevant organisms occupy a small portion of the taxonomic space. Programs such as Kraken are also highly parameter dependent and can result in spurious classifications [11]. In this work, we first show that 12mer genomic quasi-primes differentiate between closely related *Staphylococcus* spp. We then evaluate our algorithm on real-world patient data and show that by limiting our sequences to 17mer genomic quasi-primes, we achieve sensitive and specific classification of septic vs healthy patients.

## Materials and methods

### Overview

We first provide an overview of our methods. We defined our organisms of interest (Supplemental Data), including common causes of sepsis as well as the human genome [12]. We acquired the genomes from NCBI Datasets [13,14]. Representative organisms were chosen based on a list of infectious diseases found at https://emedicine.medscape.com/infectious_diseases.

To choose an appropriate kmer size, we considered a balance of sequence specificity and performance. The average number of occurrences of a kmer in a sequence is equal to the length of the genome divided by ${\text{4}}^{k}$. For a single 5’-3’ sequence of length L, there are L−k+1 contiguous subsequences of length k. Since k≪L, we will assume that there are L such kmers. Since there are ${\text{4}}^{k}$ possible kmers and the L kmers are distributed among these, the average number of occurrences of each kmer in the sequence is $L/{\text{4}}^{k}$. If we also consider the reverse complement of the sequence, the sequence length is effectively doubled, though we ignore this fact in this estimation. To have an average of one occurrence per kmer, we must therefore have that


$$\begin{array}{c} L\approx {\text{4}}^{k}\to k\approx {\text{log}}_{\text{4}}L. \end{array}$$ (1)

This argument generalizes to the case of multiple sequences and chromosomes, where L becomes the total number of bases across all nucleotides.

Using Eq (1) as a rough guide, for our proof-of-concept classification of *Staphylococcus* spp., the combined length of the staphylococcal genomes was 23,039,377 base pairs. Since ${\text{log}}_{\text{4}}\text{2}\text{3},\text{0}\text{3}\text{9},\text{3}\text{7}\text{7}\approx \text{1}\text{2}$, we chose to classify 12mers for this first analysis. For the sepsis analysis, the human genome alone was over 3 billion bases in length. Since ${\text{log}}_{\text{4}}\text{3}\times {\text{1}\text{0}}^{\text{9}}\approx \text{1}\text{6}$ and the remainder of the genomes which we included in sepsis classification contributed a billion or so more bases, we chose to classify 17mers for the sepsis analysis.

Finally, we created a master table, in which each 17mer was mapped to its corresponding species (Fig 1C). Since each 17mer was encoded in base 4 and we chose fewer than ${\text{2}}^{\text{8}}=\text{2}\text{5}\text{6}$ species, this resulted in a byte vector of length ${\text{4}}^{\text{1}\text{7}}$, with each entry being the species ID encoded as a byte. The result was a dictionary that fits within 17.2 GB of RAM and is highly accessible to consumers. With this dictionary, our method classifies 2 billion 17mers in 20 minutes on a single core of a 2.2 GHz Xeon E5-2650v4, with a processing speed of several million bps / second. Additionally, we parallelized the various tasks in our pipeline, accelerating the construction of the master table and classification of samples. Our pipeline is implemented in Julia, and we call our package Gprime.jl.

### Shotgun metagenomic sequencing of *Staphylococcus aureus* in a background microbiome

*S. aureus* ATCC 12600 (type strain, alias NCTC 8532, isolated from pleural fluid) purchased from the ATCC was selected as the target species, and eight other *Staphylococcus* species were used as the background microbiome (Table 1). These isolates were cultured on BHI agar for 16–18 hours. Using 10 µL disposable loops, four loops of overnight cultured cells were collected and transferred to tubes containing beads and 500 µL of PBS. The tubes were then horizontally vortexed, followed by centrifugation. Two hundred microliters of the supernatant were subsequently used for gDNA extraction via the QIAGEN QIAamp DNA Blood Mini Kit. The extracted DNA from these isolates was adjusted to the same concentration (e.g., ~ 120 ng/µL). The adjusted DNA from eight other *Staphylococcus* species were included, mixed and treated as in the background microbiome to assess the specificity of quasi-primes for the detection of *S. aureus* DNA sequences in a test sample that included *S. aureus* and the background microbiome, combined in a 1:1 ratio. *S. aureus* DNA was tenfold diluted in background microbiome DNA (0:1, 1:1, 1:10, 1:100, and 1:1000) to assess the limit of detection of Nanopore sequencing for the detection of *S. aureus* DNA in background microbiome DNA.

**Table 1:** *S. aureus* and 8 other *Staphylococcus* species included in the limit of detection experiment.

Isolate Number	Species	Source of isolation	Target or background microbiome	Genome Size
PS03046	*S. aureus* (ATCC12600)	Pleural fluid	Target	2.8 Mbp
PS01783	*S. epidermidis*	Ready-to-eat food product	Background microbiome	2.5 Mbp
PS01400	*S. hominis*	Raw milk	Background microbiome	2.3 Mbp
PS01871	*S. capitis*	Raw milk	Background microbiome	2.5 Mbp
PS01480	*S. equorum*	Raw milk	Background microbiome	2.8 Mbp
PS01395	*S. warneri*	Raw milk	Background microbiome	2.5 Mbp
PS01784	*S. cohnii*	Dairy powder	Background microbiome	2.7 Mbp
PS01910	*S. pasteurii*	Whey protein isolate liquid	Background microbiome	2.5 Mbp
PS01361	*S. haemolyticus*	Bovine manure	Background microbiome	2.6 Mbp

### Acquisition of human data

We used the NCBI Datasets to acquire all the sequencing data. We extracted the 17mers using Jellyfish 2.3.0 [15]. For the human genome, we used the T2T-CHM13 version 2.0 [12] assembly. We examined the metadata for each study to determine how septic vs healthy patients were encoded. For each accession number, we downloaded and parsed the XML summary of all sequences from NCBI BioSamples. The list of genomes by accession number that were used can be found in our Supplemental Data on Zenodo (publication/data/genomes_downloaded.txt).

Septic vs healthy patient datasets were also acquired from NCBI BioProjects, with accession numbers as follows: PRJEB13247 [16], PRJEB21872 [17,18], PRJEB30958 [18], and PRJNA647880 [19]. The first three accessions originated from the same institution/research group, which we refer to as Fraunhofer (18 healthy samples and 305 septic samples). The last accession is Herwanto (40 healthy and 39 septic). All three Fraunhofer accessions consisted of cell-free DNA isolated from plasma, sequenced using the Illumina HiSeq 2500 platform. Herwanto consisted of cell-free RNA isolated from peripheral blood, sequenced using the Illumina HiSeq 4000 platform.

### Identification of quasi-primes and creation of the master table

First, for each genome, we identified all subsequences of length k  (i.e., the kmers) in that genome. For example, let genome 1 be ATCGGC. Then the set of 3mers $G_{\text{1},\text{3}}$ is {ATC, TCG, CGG, GGC}. If the set of 3mers for genome 2 were $G_{\text{2},\text{3}}$ = {ATC, CCC, GGA}, then the 3mers specific to genome 1 and not included in genome 2 would be {TCG, CGG, GGC}. If we had more genomes, we would similarly exclude 3mers in genome 1 which were contained in those other genomes, until finally we have only those 3mers in genome 1 which are not contained in any other genome, which we would call $G_{\text{1},\text{3}}^{\prime }$. In set notation, we perform a set subtraction between $G_{\text{1},\text{3}}$ and the union of all other 3mer sets ${⋃}_{j\neq i}G_{j,\text{3}}$: $G_{\text{1},\text{3}}^{\prime }=G_{\text{1},\text{3}}\;\backslash \cup_{j\neq i}G_{j,\text{3}}$.

For species s with multiple genome assemblies available ${\{s}_{n}\}$ (such as for the various strains of *E. coli*), we merged the set of kmers across all such genome assemblies: $G_{i,k}=\cup_{n}s_{n}$. Once we calculate all of the $G_{i,k}^{\prime }$, we create a master table M , which maps each sequence in $G_{i,k}^{\prime }$ to i . We interpreted each sequence as a number in base 4, representing A⟺0,G⟺1,C⟺2,T⟺3 . For example, a sequence ATCG would be encoded as ${\text{0}\text{3}\text{2}\text{1}}_{\text{4}}=\text{0}\times {\text{4}}^{\text{3}}+\text{3}\times {\text{4}}^{\text{2}}+\text{2}\times {\text{4}}^{\text{1}}+\text{1}\times {\text{4}}^{\text{0}}=\text{5}\text{7}$ in base 10. The map M  can thereby be represented as an array of size 4^k^. If the preceding sequence (ATCG) corresponds to species 7, then the 57^th^ entry of M is set to 7.

For 17mers, M  is an array of size ${\text{4}}^{\text{1}\text{7}}=\text{1}\text{7},\text{1}\text{7}\text{9},\text{8}\text{6}\text{9},\text{1}\text{8}\text{4}$. The data type of the entries can be optimized to the number of species. For example, a byte can represent an unsigned integer from 0 to 255. If we have at most 256 species, then M can be efficiently represented as an array of bytes of length ${\text{4}}^{\text{1}\text{7}}$, corresponding to exactly ${\text{4}}^{\text{1}\text{7}}$ bytes (≈17 gigabytes) of memory.

### Classification and normalization of read counts

We performed classification of 17mers for each sample via Jellyfish [15] and the master table, resulting in a vector of read counts for each sample. To normalize the read counts, we first divided all read counts by the total number of reads. Next, for all the samples (Herwanto + Fraunhofer), we centered the normalized read counts by subtracting the mean and dividing by the standard deviation. By performing these two operations in this order, we retained the distributional information of each species’ read count in the context of the sample’s run characteristics before standardizing the inputs to regularize our logistic regressions.

### Resampling Fraunhofer

Whereas the Herwanto dataset was appropriately balanced between healthy and septic samples (40 and 39, respectively), only 5.6% (18 of 323) of the Fraunhofer dataset were from healthy patients. Such imbalanced data easily lead to overfitting [20]. As a reasonable starting point, we assume that the prior probability that a given sample is septic is 50%, i.e., the clinician is 50% sure that the patient is septic. To do this, we combined the 18 healthy Fraunhofer samples with 18 samples chosen uniformly at random from the remaining 305 septic Fraunhofer samples. We performed this undersampling repeatedly to estimate the empirical distributions of our performance metrics.

### Logistic regression

We used scikit-learn 1.5.1 [21] to perform logistic regressions (Figs 1E-F), with hard-coded random seeds for reproducibility. The Herwanto and undersampled Fraunhofer datasets were stratified by healthy vs septic status and uniformly randomly split into training and test sets at a ratio of 3:1, except as noted in the code.

Logistic regressions are used for binary classification tasks, such as classifying the reads in this work as healthy vs septic. Logistic regressions require the fitting of the weights $a_{\text{0}},a_{\text{1}},\cdots ,a_{n}$ in


$$p(x)=\frac{\text{1}}{\text{1}+e^{-a_{\text{0}}+\sum_{\text{i}}a_{i}x_{i}}},$$

where the $x_{i}$ are the input features (i.e., the normalized read counts), p(x)=1  represents the positive class, p(x)=0 represents the negative class, and the coefficients $a_{i}$ are fit so that the binary log-loss L (or cross-entropy loss) is minimized:


$$\begin{array}{c} L=-\sum_{x}\left[y(x)\text{ln}p(x)+\left(\text{1}-y(x)\right)\text{ln}\left(\text{1}-p(x)\right)\right], \end{array}$$

where x are the inputs taken from the training set, y(x) is the true label of the sample (septic vs healthy), and p(x) is the output of the logistic regression from above. An additional regularization term was added to L to help mitigate overfitting:


$$\begin{array}{c} L_{reg}=\frac{\text{1}}{C}\sum_{\text{i}}a_{i}^{\text{2}}, \end{array}$$

so that the total loss to be minimized was $L_{total}=L+L_{reg}$. Finally, we also performed a hyperparameter search, resulting in a relative penalty factor of C = 100 for the L^2^ logistic weight regularization and 50 training iterations.

### Statistics

Linear regressions with associated significance testing were performed using SciPy [22]. Hotelling’s T^2^-test [23] was performed using the pingouin package [24].

### Performance metrics

The logistic regression produces numbers in the range [0,1], which can be interpreted as the probability that a given input belongs to the positive class. By varying the threshold for these probabilities to be considered positive vs negative, we may characterize the classification performance of the logistic regression. The classification performance metrics we examined were the receiver operating characteristic (ROC) curve and its associated area under the curve (AUC) (Figs 1G-H), as well as the precision‒recall (PR) curve and its associated average precision (AP) (Figs 1I-J). These quantities were calculated using the scikit-learn package [21].

For a given cutoff, logistic regression yields rates of true positives (TPs), false positives (FPs), true negatives (TNs), and false negatives (FNs), which are used to calculate performance metrics. The ROC is a plot of the TP as a function of the FP, and the AUC of the ROC is computed via the trapezoidal rule on the ROC curve. For the PR curve, the precision P and recall R are defined as follows:


$$P=\frac{TP}{TP+FP},$$

$$R=\frac{TP}{TP+FN}.$$

The AP is defined as:


$$AP=\sum_{\text{n}}(R_{n}-R_{n-\text{1}})P_{n},$$

where ${(R}_{n},P_{n})$ are the recall and precision, respectively, calculated at the thresholds. Note that this definition differs from the trapezoidal rule for the AUC curve.

To estimate the median AUC, median AP, and 95% confidence intervals, we performed bootstrapping by repeatedly resampling Herwanto and Fraunhofer into training and test sets (Figs 1G-J). The distributions, medians, and 95% confidence intervals in Figs 1G-J were calculated from 1000 bootstrap samples, whereas only the first 100 of the corresponding AUC/PR curves were plotted for ease of visualization.

To evaluate the importance of each species to the logistic regression, we trained 100 logistic regression models with random, stratified splits of Herwanto and Fraunhofer and counted the number of times a given feature had nonzero permutation feature importance (Figs 1K-L). All calculations are shown in the included Jupyter notebooks [25] (Supplemental Data).

## Results

First, to validate quasi-prime read assignment, we used shotgun metagenomic sequencing of DNA from *Staphylococcus aureus* combined at varying concentrations with eight other *Staphylococcus* species. Using reference assemblies for these species we determined their 12mer quasi-primes. As noted in the Methods, we chose a length of 12 for this proof-of-concept analysis to match the number of possible 12mers to the total size of the staphylococcal genomes, so that each 12mer corresponds to an average of a single species. We classified the reads for each sample and plotted the read proportion as a function of the initial *Staphylococcus aureus* concentration (Fig 1D). The concentrations were logarithmically spaced, and thus, the linear regressions appeared exponential. There was excellent agreement between the classifications and *Staphylococcus aureus* concentrations (p = 1.9e-5). These results demonstrate the ability of our approach for distinguishing bacterial strains in multi-culture experiments.

Second, we used human data from NCBI Datasets which labeled septic versus healthy patients, as described in the Methods section. Blood culture data were unavailable. We performed principal component analysis on the normalized read count data, plotting Herwanto (cell-free RNA) versus Fraunhofer (cell-free DNA) (Fig 1E). We found that the Herwanto data (Fig 1F) were clearly distinct from the Fraunhofer data (Hotelling’s T^2^ = 6080.3, p < 10^−10^). Therefore, subsequent analyses of the Herwanto and Fraunhofer datasets were performed independently.

For simplicity and interpretability, we used logistic regressions to predict whether the patient would be septic or healthy in both datasets. For Herwanto, we performed repeated random splitting of the points between the training and test sets. Owing to the severe data imbalance in Fraunhofer, we performed repeated undersampling of that dataset (Methods). We plotted the distribution of AUCs and the associated receiver operating characteristic (ROC) curves (Figs 1G-H). We found that the median AUCs for the datasets were high (approximately 0.9 for both), indicating high sensitivity and specificity. We then examined the distribution of average precision and the associated precision‒recall curves (Figs 1I-J); the median average precision was similarly high (Herwanto: 0.877, Fraunhofer 1.0).

We examined the most important factors (Methods) for each logistic model (Figs 1K-L), which revealed that the *Homo sapiens* count was near the top in importance for both datasets. The Fraunhofer dataset emphasized other common sepsis pathogens, such as *Pseudomonas*, *Enterococcus*, and *Haemophilus*.

Finally, we performed additional analyses using Kraken 2, which are included as Supporting Information (Supplemental Methods, S1-S3 Figs). We find that the full (~60–70 GB) database achieved similar performance to our method when the output was restricted to the species we tested, whereas the truncated (~16 GB) and custom-built Kraken 2 databases did not produce sufficient read assignments to classify septic vs healthy patients.

## Discussion

A major predictive factor of sepsis was the number of reads classified as human, presumably because a lower proportion of reads classified as human indicates increased reads classified as exogenous. Common etiologies of sepsis, including *Pseudomonas*, *Enterococcus*, and *Haemophilus*, were identified [16–19,26].

Due to the small number of taxa that can thrive in the human body, we may limit the size of the dictionary of quasi-G-primes significantly compared with that of universal taxonomic classifiers such as Kraken 2 with its standard databases. Furthermore, organisms belonging to similar taxa are often treated with the same set of standard broad-spectrum antibiotics. For example, Gram-negative organisms are often treated with third- or fourth-generation cephalosporins, while Gram-positive organisms and methicillin-resistant *Staphylococcus aureus* are treated with vancomycin, and thus ceftriaxone/cefepime + vancomycin is a common treatment of choice [27,28]. A potential diagnostic benefit of our method’s sensitivity is that it may become possible to rule out significant bacteremia via next-generation sequencing and thereby improve antimicrobial stewardship for patients. However, further validation with blood cultures is necessary to assess the clinical accuracy of our method in identifying the causative organism in sepsis. The major weakness of this work and of the literature is the paucity of blood-sequenced sepsis cases with known organisms due to the poor sensitivity of the gold standard of blood culture for diagnosis; therefore, we were limited solely to sepsis/healthy classification.

There is a need for improved normalization of experimental techniques between research groups and NGS techniques, as indicated by the heterogeneity seen in the principal component analysis. The cell-free DNA sequencing of the Fraunhofer dataset potential provides a less biased view of the microbiome population, compared to the cell-free RNA sequencing of the Herwanto dataset. However, we were able to predict septic versus healthy patients in both populations in separate logistic regressions, indicating that there is sufficient signal in both types of sequencing for classification. Our importance analysis was limited due to the low number of healthy controls in Fraunhofer. In the Supporting Information (S1 File, S1 Fig, S2 Fig, S3 Fig), we demonstrate that our method is more memory efficient, sensitive, and accurate than Kraken 2.

There are many strains of specific bacterial species, which can significantly affect the availability of quasi-G-primes. For example, in this work, we performed a union on all strains of *Staphylococcus aureus*, which may be inappropriate for methicillin-sensitive vs methicillin-resistant strains, where the clinical question is whether to use MRSA-targeted therapies. It may therefore be beneficial to focus on plasmid-associated kmers which confer specific antibiotic resistance in such cases.

In this work and in our other recent publications [3–5], there is a focus on quasi-primes because they are specific to a single species. With quasi-G-primes, one requires |G|−1 kmers to differentiate among all |G| species in a dataset with perfect specificity. In principle, only $\lceil {\text{log}}_{\text{2}}|G|\rceil$ specially selected kmers are needed for 100% specificity; in the case that the first kmer divides G into two halves, A and B, the second kmer perfectly divides A and B into four quarters, and so on. In practice, sequencing is a stochastic and noisy process; thus, many more quasi-G-primes were used in this work to increase its sensitivity. Furthermore, our current selection of species was informed by clinical relevance, and in our future work we plan to encompass all available species to further refine our quasi-G-prime lists.

In future work specific to sepsis, it may also be beneficial to group bacterial taxa or strains by the class of antibiotics which are generally most effective and search for kmers that differentiate between those groups of bacteria, enabling the narrowing of antibiotics in the hospital setting. There is a spectrum of specificity to kmers, from quasi-G-primes specific to a single organism to the gestalt genomic fingerprints considered in microbiomics, all of which may be useful in the treatment of sepsis.

The simple idea of genomic quasi-primes enables highly sensitive and specific determination of species, especially when the plausible taxonomies are limited. Future applications of quasi-primes in personalized medicine may even use the patient’s own genome in place of the generic human genome as a control, to increase detection of foreign genomic material. Our method is effective and has potential for practical application in biology and medicine. By varying the definition and grouping of taxa, future work may achieve different classifications, which may inform clinical practice.

## Supporting information

S1 Fig Full Kraken 2 database analysis. Applying the full Kraken 2 database (~60–70 GB RAM) to the same benchmarking tasks as provided in the main text. **A-I** correspond to subfigures **1D-L** in the main text. The results are largely similar to that of our method, at the cost of RAM and additionally some lower accuracy and specificity for the Herwanto dataset.
(SVG)

S2 Fig Truncated Kraken 2 database analysis. Applying the truncated Kraken 2 database (~16 GB RAM) to the same benchmarking tasks as provided in the main text. **A** shows the benchmarking of this database against cultured *Staphylococcus* spp. When restricted to the list of sepsis-relevant genomes, this truncated database did not generate any counts for the sepsis species in septic patients, and we were unable to plot those results.
(SVG)

S3 Fig Custom Kraken 2 database analysis. Applying the custom Kraken 2 databases to the same benchmarking tasks as provided in the main text. **A-I** correspond to subfigures **1D-L** in the main text. In **A**, the database was built only upon the *Staphylococcus* species which were cultured. Notably, Kraken 2 did not pick up any counts for *Staphylococcus warneri*. The sepsis classification task was similarly difficult for the custom database (**B-I**), with few septic samples demonstrating any read counts.
(SVG)

S1 File Supplemental Methods and Discussion. (DOCX)
