# Supplementary material for: Doing more with less: Genomic quasi-G-primes differentiate septic from healthy patients
Source: PLoS One. 2026 Feb 6;21(2):e0341828. doi: 10.1371/journal.pone.0341828 (PMC12880686; doi:10.1371/journal.pone.0341828)
Supplement: S1 File — (DOCX) [file pone.0341828.s004.docx]

## Supplemental Methods

To evaluate the efficacy of the quasi-prime method as compared to the existing Kraken 2 framework in detecting sepsis, we used various versions of Kraken 2 databases on the same data [(Wood, Lu, and Langmead 2019)](https://paperpile.com/c/aMkbij/LKht).

The first Kraken 2 database was the “PlusPF” database dated June 5 2024, available at (<https://benlangmead.github.io/aws-indexes/k2>), which represents a comprehensive database of available Refseq archaea, bacteria, viral, plasmid, human, protozoa, and fungi genomes. We call this version simply Kraken.

The second Kraken 2 database was the “PlusPF-16” database dated June 5 2024, available at the same website, which is a memory-limited version of the full database to 16 GB. We chose this database to provide an additional comparison with the same amount of RAM required as our method.

The third Kraken 2 database was a custom database using default settings which we built using the NCBI taxonomy and the same genomes that we included in each benchmarking task. For the *Staphylococcus* benchmarking task, we built the custom database using only the *Staphylococcus* species which were cultured. For the sepsis classification task, we built the database with the sepsis species genomes that were used in our quasi-G-prime method.

With the first two versions of the Kraken 2 database (which include all genomes available), we restricted the resulting counts to the short list of organisms which we focused upon in this work, as sepsis-relevant species. For all three methods, we used Bracken [(Lu et al. 2017)](https://paperpile.com/c/aMkbij/ZoI2) to refine counts based on statistical noise, which is the recommended method for interpreting the results of Kraken 2.

## Supplemental Discussion

We found that the full version of Kraken 2 database with all Refseq genomes reproduced our results for both the *Staphylococcus* species culture task and sepsis classification task, with the exception of a lower sensitivity and specificity (Figure S1-D, S1-F) for the Herwanto dataset. We found that the truncated database (Figure S2-A) performed the *Staphylococcus* species culture task well, but was unable to identify any of the same sepsis pathogens as the full database or our quasi-G-prime method. When we built custom Kraken 2 databases with default settings, we found that Kraken 2 classified 0 reads as *Staphylococcus warneri*, in contrast to all other methods presented.

First, it is reassuring that the full Kraken 2 database reproduces our results (Figure S1). However, our method possesses a significant advantage over Kraken 2 in terms of RAM (17 GB vs 70 GB) usage, enabling easier access to sepsis read classification on consumer hardware, and lowering the barrier for implementation in the hospital.

Second, it is apparent that truncating the full database to lower computation resource usage is not a viable strategy, as all of the sepsis species were assigned counts of 0 in the 16 GB version of the Kraken 2 database (Figure S2).

Third, building a custom Kraken 2 database resulted in 0 counts for one of the *Staphylococcus* species (Figures S3-A through I), and was similarly ineffective in the sepsis classification task as the truncated full database (Figure S3. In the *Staphylococcus* task, we used solely a single reference genome for each of the cultured species to build the quasi-G-prime database as well as the Kraken 2 database. The 0 counts for *Staphylococcus warneri* in the custom database versus in the full database can therefore be explained by the inclusion of non-reference genomes in the full database. For the sepsis classification task however, many non-reference genomes for each species were included, and therefore the custom Kraken 2 sepsis database is inferior to the quasi-G-prime approach.

We remark that some of the apparent inadequacies of the Kraken 2 approach may be due to non-optimal choice of settings, however we emphasize that we used the default settings and Bracken pipeline in order to provide a fair out-of-the-box comparison. Optimizing Kraken 2 settings is outside the scope of this work. Kraken 2 uses a hashmap to classify sequences, and so there can be key collisions which result in misclassification of sequences (the Kraken 2 authors estimate <1% chance of this occurring). Kraken 2 also uses a default kmer size of 35, which increases the specificity of classification at the cost of sensitivity, whereas we used a kmer size of 17. When the sensitivity is sufficiently low due to the use of long kmers, misclassifications of 1% of reads can become significant, and thus the Bracken pipeline was introduced to mitigate statistical errors and false positives.

In terms of human disease, there are thought to be very few taxa relative to the tree of life which are capable of (1) replicating in humans and (2) can cause significant pathology. Therefore, while Kraken 2/Bracken may be suitable for classification of diverse organisms such as in microbiome studies, it is not necessarily appropriate for a focused task such as differentiation of highly-related sepsis taxa. Even though the sepsis taxa are highly related, crucial differences in antibiotic resistance such as those conferred through plasmid exchange can make a significant difference in optimal antibiotic choice in sepsis. We highlight again that the quasi-G-prime approach is a conceptually elegant, sensitive, and specific approach to such tasks.

## References

[Lu, Jennifer, Florian P. Breitwieser, Peter Thielen, and Steven L. Salzberg. 2017. “Bracken: Estimating Species Abundance in Metagenomics Data.” *PeerJ Computer Science* 3 (January):e104.](http://paperpile.com/b/aMkbij/ZoI2)

[Wood, Derrick E., Jennifer Lu, and Ben Langmead. 2019. “Improved Metagenomic Analysis with Kraken 2.” *Genome Biology* 20 (1): 257.](http://paperpile.com/b/aMkbij/LKht)
